# A qualitative examination of the perceptions of parents on the Canadian Sedentary Behaviour Guidelines for the early years

**DOI:** 10.1186/1479-5868-11-65

**Published:** 2014-05-17

**Authors:** Valerie Carson, Marianne Clark, Tanya Berry, Nicholas L Holt, Amy E Latimer-Cheung

**Affiliations:** 1 Faculty of Physical Education and Recreation, University of Alberta, Edmonton, AB, Canada; 2 School of Kinesiology, Queen’s University, Kingston, ON, Canada

**Keywords:** Young children, Parents, Sedentary behavior, Guidelines

## Abstract

**Background:**

Minimizing sedentary behavior, in particular screen-based sedentary behavior, during the early years is important for healthy growth and development. Consequently, new Canadian Sedentary Behaviour Guidelines for the Early Years (aged 0–4 years) were recently released. Researchers are unclear what messages should supplement the guidelines when disseminating them to parents and when using the guidelines in behaviour-change interventions to increase adoption. The objective of this study was to qualitatively examine parents’ perceptions of the new Canadian Sedentary Behaviour Guidelines for the Early Years.

**Methods:**

Parents with a child ≤4 years who attended a child care centre were purposefully recruited from child care centres. A total of 7 semi-structured focus groups with 2 to 5 parents were conducted from August to November, 2013 by a trained and experienced moderator. Participants were asked a series of open-ended questions pertaining to the Sedentary Behaviour Guidelines information sheet. Initial themes were identified followed by further review and analysis.

**Results:**

For the most part parents thought the guidelines were clear and did not disagree with the recommendations per se. However, some confusion arose around the value of some sedentary activities, such as reading and coloring, for social and cognitive development. Many parents described feeling guilty after reading the guidelines and perceived several barriers in meeting the daily recommendations. Common barriers included the need to balance multiple demands of family life, the prevalence and accessibility of screen technology, and the weather and built environment where families live. Parents expressed the importance of communicating the guidelines early enough for good habits to be established and the need for realistic strategies and ideas to help them meet the recommendations.

**Conclusions:**

Overall the findings indicate that gain-framed messages around the role of screen-based and non-screen-based sedentary behavior for children’s cognitive and social development might be most effective for adoption of the guidelines. Furthermore, providing parents the guidelines early with resources for minimizing sedentary behavior should also be considered. Future research is needed in other demographic groups of parents to confirm these findings.

## Introduction

The early years (≤4 years old) are the most critical period of overall development throughout the lifespan. In particular, the early years are marked by an intensive period of brain development [1]. Accumulating evidence indicates that minimizing sedentary behavior, in particular screen-based sedentary behavior like television viewing, during the early years is important for healthy growth and development [2]. For instance, recent systematic review evidence found unfavourable associations between screen time and a variety of health outcomes, including adiposity (e.g., body mass index, skinfolds), psychosocial health (e.g., aggressive behavior, self-control, social skills, cooperation), and cognitive development (e.g., attention, vocalization count, reading recognition/comprehension, memory scores) among young children aged ≤4 years [2].

Until recently, the study of sedentary behavior in young children was largely neglected [3]. This was primarily due to the assumption that young children are naturally active [4]. However, research indicates that young children today spend a significant portion (>73%) of their waking hours sedentary [5]. The vast majority of children (80%) are beginning to watching television before the age of 2 years [6,7]. Once these screen time habits are formed they tend to track over time [8,9] and predict health outcomes in adulthood [10]. Therefore, the early years represent an important period for establishing appropriate sedentary behavior habits for both current and later health [10].

Since parents are fundamental in children’s socialization and have major influences on children’s development [11], parents’ role in shaping and maintaining young children’s sedentary behavior habits is important to consider. This concept is supported by a recent study that found parental cognitive factors were the primary predictors of young children’s screen time including, positive attitudes towards screen time (*r* = 0.62), barriers to reducing screen time (*r* = 0.54), perceived norms around screen time (*r* = 0.58), and self-efficacy to establish boundaries for screen time (r = −0.26). These factors explained 38% of the variance in screen time among children aged ≤5 years and 42% of the variance among children ≤3 years [12]. Previous qualitative work in pre-school children (aged 3–5 years) indicates parents are typically not concerned about their child’s screen time [13,14]. In fact, many parents have positive attitudes towards their child’s screen time engagement, especially for learning [13-15] and socializing [16]. Evidence indicates the best way for children to learn is through child-caregiver interaction [2,17]. However, screen time tends to reduce child-caregiver interaction and for younger children, under the age of 2 years, screens are thought to be particular detrimental to cognitive development due to brain overstimulation [18].

Given the importance of establishing appropriate sedentary behavior habits in the early years, recent systematic review evidence [2] was used to help inform the development of the first Canadian Sedentary Behaviour Guidelines for the Early Years (aged 0–4 years), which were released by the Canadian Society for Exercise Physiology in March, 2012 [3]. For healthy growth and development, the Canadian Sedentary Behaviour Guidelines for the Early Years recommend minimizing the total time spent being sedentary in a day including prolonged sitting or being restrained (e.g., stroller, high chair) for more than one hour at a time. Further, for children under 2 years, screen time is not recommended and for children 2–4 years, screen time should be limited to under 1 hour per day [3]. Similar recommendations exist in other countries, such as Australia [19], United Kingdom [20], and the United States [21]. National surveillance data indicate that only 18% of Canadian 3–4 year olds met the screen time recommendations of the guidelines [22]. Similarly, only 32% of children aged <2 years from a local sample in Ontario, Canada met the recommendations [6]. Similar findings have been observed in other countries where recommendations exist. Therefore, efforts are required to increase the adoption of the Sedentary Behaviour Guidelines.

During the guideline development process a need was identified for future research on the most effective ways to communicate the new guidelines to intermediaries such as parents [4]. To date, no study has examined parents’ perceptions of the Sedentary Behaviour Guidelines and barriers to meeting them. Addressing this research gap can help determine what messages should supplement the guidelines when disseminating them to parents [23,24] and when using the guidelines in behavior-change interventions to increase adoption. Furthermore, this knowledge can inform the next update of the Canadian Sedentary Behaviour Guidelines for the Early Years scheduled for 2016 [3]. Therefore, the objective of this study was to qualitatively examine parents’ perceptions of the new Sedentary Behaviour Guidelines for the Early Years.

## Methods

### Participants

Parents with a child ≤4 years who attends a child care centre were purposefully recruited from child care centres that largely served professionals in Edmonton, Canada. Edmonton is a northern Canadian city characterized by extremely cold temperatures and shorter daylight hours in the winter. Parents were primarily recruited through information letters, newsletters, and presentations at parent meetings. A total of 7 semi-structured focus groups with 2 to 5 parents were conducted from August to November, 2013. Ethics approval was obtained from the University of Alberta Human Research Ethics Board. Written informed consent was obtained from all participants.

### Procedures

Following the guidelines provided by Kitzinger [25] and Morgan [26] all focus groups were facilitated by a trained and experienced moderator and lasted for 30 to 45 minutes. At the beginning of the focus group parents completed a brief questionnaire that included both child and parent demographic information. If the parent had more than one child aged 1 month to 4 years old, the questionnaire was completed for the child with the birth date closest to January 1^st^. Parents were then provided a formal definition of sedentary behavior and given the Canadian Sedentary Behaviour Guidelines for the Early Years (aged 0–4 years) information sheet to review [3]. Next, participants were asked a series of open-ended questions pertaining to the information sheet (see interview guide below). Prior to adjourning the focus groups, parents were given the opportunity to provide any further comments. The focus group questions were initially developed by the research team using the guideline information sheet as the focal point [3]. The questions were then further refined to minimize overlap and repetitiveness.

### Interview guide

1) What does sedentary behavior mean to you? Is this definition similar/different than what you thought it was?

2) How clear are these guidelines?

3) What is your initial reaction to the guidelines? How do they make you feel as a parent (e.g., Irritated/Frustrated/Happy/Guilty)?

4) Why do you think these guidelines are feasible/unfeasible or realistic/unrealistic?

5) The guideline sheet lists several health benefits for young children associated with meeting the guidelines. What are your thoughts about these statements? Do you agree or believe them?

6) From what source would you need this information from to consider it credible/trustworthy?

7) How confident are you that your child could meet these guidelines? What barriers do you see?

8) How would you put the guidelines into practice? Are the suggestions on the bottom of the guideline sheet helpful?

9) How could the guidelines best be communicated/presented to parents?

10) How could these guidelines be presented so that they were most helpful to you?

### Data analysis

All focus groups were audio recorded and transcribed by a professional company. Data analysis began as soon as the data from the first focus group were transcribed. Following guidelines from Stewart et al. [27], the first transcript was read several times by the moderator and lead researcher to identify sections relevant to the research objective. This process was repeated for all transcripts. The moderator and lead researcher met regularly to discuss the analysis and developed a classification system of themes reflecting the major topics and issues in the data. All relevant data (e.g., phrases, sentences, or exchanges between individual respondents) from the transcripts were placed into these themes. A ‘long list’ of initial themes was created, which was then subjected to further review and analysis among all members of the research team. Ultimately, six themes that addressed the research objective were agreed upon after the analysis of the sixth focus group. The seventh, and final, focus group was then completed and data were coded into these six themes. At this point it was agreed upon by the research team that an adequate amount of data saturation had been obtained because each of the themes had sufficient detail and variety [28].

## Results

Participant characteristics of the 27 parents from the 7 focus are in Table 1. The mean age of the child with a birth date closest to January 1^st^ (some families had multiple children in the ≤4 age range) was 33.5 (SD 14) months. Approximately 30% of those children had a younger sibling and approximately 33% had an older sibling. The mean age of the participating parent was 36 (SD 6) years and the majority of parents were mothers (85%) who were married (89%), and had a graduate degree (74%). This indicates the focus groups included homogenous groups of parents [25,26].

**Table 1:** Participant characteristics

	**Total (n = 27)**
Child age (months)	33.5 (13.5)
Child sex	
Male	40.7
Female	59.3
Young siblings	
No	70.4
Yes	29.6
Old siblings	
No	66.7
Yes	33.3
Parent age (years)	36.3 (5.8)
Parent gender	
Male	14.8
Female	85.2
Marital status	
Married	88.9
Living common-law	11.1
Parental education	
Community/Technical College	11.1
Undergraduate degree	14.8
Graduate degree	74.1

Data presented as mean (standard deviation) for continuous variables and % for categorical variables.

### General impressions about the guidelines

Feedback from parents indicated that the guidelines were generally clear and easy to understand. Parents expressed support for the overall intent of the guidelines and largely considered the information to be helpful. However, some ambiguity was noted around the value of some sedentary activities, such as reading, coloring, and arts and crafts. One parent stated, “Reading is a sedentary activity, and I think reading does enhance learning” (FG4). Parents also experienced feelings of guilt after reading the guidelines and described barriers that prevented their children from meeting the daily recommendations. For example, one parent lamented, “I felt bad right away [after reading the guidelines]. I know my kids sometimes watch more TV than that, because I need to get stuff done. So I immediately felt bad” (FG6). Barriers reflected the particularities of the social and physical context in which families live including, the need to balance multiple demands of family life, the prevalence and accessibility of screen technology, the necessity of commuting long distances for work and child care, and the practicalities associated with living in a cold climate.

### Is all sedentary time created equal?

Parents generally agreed with the overarching ideas presented by the guidelines and appreciated the specificity of recommendations. However, there was widespread concern and confusion that these guidelines painted all sedentary behavior as ‘bad’. Many parents felt strongly that sedentary behaviors, such as reading and colouring, were not harmful; rather, they were critical for their child’s development. Some felt strongly that their children needed the calmness afforded by engaging in these behaviors, particularly if they were at child care all day or in otherwise stimulating environments. At times, parents questioned the status of creative activities as sedentary and therefore as ‘bad’ according to these guidelines.

These [guidelines] are a bit confusing because right now I’m trying to train my daughter, she’s three and a half, to learn some stuff, so sometimes to calm her down we sit at the table and I’ll teach her something… putting puzzles together, stuff like that. I think that’s one way to train focus and attention. But here it says that’s not good…” (FG2).

Another parent was similarly confused, well is sitting and colouring really a sedentary behavior? Or playing with Play-Doh?…I think those creative activities are important” (FG3).

In addition, some parents considered television and screen time to hold some positive benefits associated with learning, education, and social skill development. Participants described using educational videos for babies and young children. One mother reflected, “Not to say that iPads are really great, but my daughter does learn things from the iPad…she does puzzles, and has the memory game on there” (FG7). Another added, “…sometimes I think television is another good way for [my daughter] to learn. It has all the good programs” (FG2). However, the perception that screens are a potential learning tool was not universally shared. Some parents agreed that interaction with the caregiver is the best learning tool, as the evidence suggests [2]. As one mother put it, “If I bring my laptop out and let [my daughter] play…the amount that she talks to me just plummets. So I can only imagine that if that’s a regular habit we’re not interacting, we’re not building vocabulary…” (FG3). Therefore, there were some tension and ambiguity around the value of sedentary activities. It was quite evident that parents greatly valued their children’s cognitive and social development. “And to me that’s probably the biggest thing, even more so than body weight, is the social skills, the intention, the desire to explore and learn” (FG3).

### Sometimes it’s just not possible: We need the ‘electronic babysitter’

Overall, parents considered the guidelines to be unfeasible in their totality. One of the most prominent barriers cited was that of needing children to engage in sedentary behavior (especially screen time) so that parents could accomplish daily tasks. Meal preparation and household chores were among those tasks most commonly discussed. One parent explained, “When my husband’s working, I’ve got three kids and I’m trying to make supper, so [the television] is my babysitter…” (FG1). Parents expressed that it would be challenging to complete important daily tasks without utilizing screen time or other sedentary behaviors. As one parent noted “It’s very tempting to let her keep watching television because I make progress in the kitchen, or I get a chance to clean the bathroom, and I always just need 15 more minutes” (FG7). Another summed it up this way, “The television for me is definitely a Nanny” (FG4).

Screen time and other sedentary behaviors (e.g., strollers) were also related to notions of safety. For example, many parents explained that putting their child in front of a child-friendly television show or keeping them in a stroller or car seat was important for keeping their child safe and occupied when direct hands-on supervision was not possible. As one mother said,

It’s a safety issue sometimes too, if I’m on my own and [my husband] isn’t around I have to have some kind of distraction for her. Because I’ve tried having her in the kitchen and we’ve ended up with burnt fingers and a lot of crying (FG3).

Similarly, one parent noted, “When they’re two and they’re running around they can be hurt, the iPad is a good way for them to be safe and you can do stuff, even for an hour” (FG4). In general, parents described feeling guilty when utilizing the ‘electronic babysitter’ but perceived few viable alternatives. However, parents were eager for specific and realistic suggestions alternative to screen time for entertaining and engaging their children when they needed to complete daily tasks.

### Tech savvy tots and the ubiquity of technology

Another important barrier to meeting the Sedentary Behaviour Guidelines was the ubiquity of technology in contemporary society. Parents explained that the prominent presence of television and electronic devices (e.g., tablets and smartphones) in the home and children’s interest and ability to use these devises made it challenging to limit screen time. As one mother said, “…my son is four and he knows the password for my phone and he can get on the Netflix app…” (FG4). Another parent agreed, “You know the 11 month-old wants to grab my phone, and she knows to look at it and push buttons” (FG7). These stories revealed that children are increasingly familiar with screen technologies at early ages. Parents also explained that television, tablets, and phones were exceptionally alluring for their children. Consequently, turning off the television or taking away the phone or tablet posed a significant challenge and was often the cause of tears and tantrums. This made limiting time on these devices even more difficult.

The importance of parental modeling also emerged strongly in discussions. Many parents described their own reliance on and use of screen technology and suggested that it was difficult to model the behavior they were asking of their children. For example, one parent said, “The kids will imitate you, so if you are on a computer they wanna be on the computer” (FG4). Another reflected, “Screen time is really challenging to avoid because it’s so naturally addictive, and as parents, if I’m a role model I’m always on my screen for my own work and for my own pleasure too…and kids see that” (FG7). Parents believed it was unfair and unrealistic to impose these regulations on their children when they were unwilling/unable to do so themselves. As one mother questioned, “How can I tell my daughter not to watch her iPad when [my husband] is watching television?” (FG4). It was quite evident that screen technology is an integral part of parents own lives, at work and within the home. Although many parents were mindful of the need to limit screen time, many conceded they struggled to achieve this due to the prominence of technology in their daily lives.

### The environment

The challenges posed by climate (cold weather) and the built environment were raised repeatedly in conversation. Parents described the difficulties associated with going outdoors (cold temperatures, the time involved in bundling children in winter wear) and the necessity of long car rides in their daily lives as barriers to achieving the guidelines. “It says take children outside every day. We do try to do it in the winter, but it’s just too cold” (FG4) said one parent. Another elaborated, “It’s a struggle to find activities to do when it’s very cold. We’re always looking, where can we go …so he can run around inside. So that’s I think a struggle living here” (FG5).

The built environment also inhibited parents’ ability to meet the recommendations in other ways. For example, the guidelines suggest that children should not be in a car seat for more than an hour at a time. However, in a large urban centre having a child in a car seat for an extended period of time is sometimes unavoidable.

I really feel that nobody wants to be driving around all the time, adults or kids. And I almost feel that by including time that they’re in the car in part of being the ‘sedentary/you’re bad’ guidelines isn’t fair because I don’t think anybody chooses to do that… (FG5).

Parents were also divided on the feasibility of breaking up long car trips with brief stops as one strategy recommended by the guidelines. Many parents had not previously considered doing this and appreciated the suggestion for the benefit of both themselves and their child. One parent described an upcoming four-hour car ride and said, “Stopping for a half an hour and trying to find a restaurant that he can run around in…that’s a terrific piece of advice” (FG1). However others adamantly suggested that it would not be feasible and would disrupt their driving routine. “We drive for seven to eight hours every other month…and there’s no way you’re getting me to stop…We are hammering it out… we are not stopping because it’s not fun” (FG4). Therefore, the realities imposed by the environment impacted parents’ abilities to meet the recommendations made by the guidelines.

### In their own words: what parents need and Want

During the focus groups, parents were invited to provide feedback about how the guidelines could be most effectively communicated to parents. Many suggested they would like to receive the information from a pediatrician. “If I had heard [about the guidelines] from our pediatrician from the very beginning then I would’ve just totally gone along with it” (FG6). Parents also indicated that receiving the information from Health Canada or Alberta Health would increase the trustworthiness of the information. Interestingly, the recommendation of zero screen time for infants less than two years was news to many parents. Participants expressed wishing they had known about this earlier, (i.e., during pregnancy) because these habits are difficult to break once they are set. As one parent described, hearing this information early makes strong impressions. “When you’re a first time Mom, you listen to everything…” (FG6).

Parents did confess that the guilty feelings of not meeting the guidelines may make them “turn off”. To overcome this, many parents suggested phrasing the information in the guidelines positively and emphasizing what parents could do, instead of what they should do and what they were doing wrong. Many parents expressed a concerted need for more specific ideas and strategies to support them and increase their capacity for meeting the guidelines.

## Discussion

This study aimed to capture and describe parents’ views on the new Canadian Sedentary Behaviour Guidelines for the Early Years that were released in March, 2012. To date, this study is the first to examine parents’ perceptions of these new guidelines. Furthermore, this study is the first to seek parents’ views of all sedentary behaviors rather than solely focusing on screen time. The findings provide important insight into how these guidelines were interpreted and received by this sample of parents as well as the barriers faced in meeting them. Furthermore, participants provided valuable suggestions on communicating and disseminating the guidelines to parents.

A novel aspect of the present study was examining parents’ interpretation of sedentary behavior. The confusion that arose for parents around reducing specific types of sedentary behaviors, such as coloring and reading, to improve children’s cognitive and social development suggests that the recommendation for children not to sit or be restrained for more than one hour at time was not clearly interpreted by parents. As a result parents appeared to perceive the guidelines to mean that all sedentary behaviors were bad regardless of the duration of engagement. This indicates supplementary messages may be needed when disseminating the guidelines to clarify the duration piece of non-screen-based sedentary behavior, such as reading and coloring. Additionally, since the systematic review evidence that helped inform the Sedentary Behaviour Guidelines was primarily based on screen-based sedentary behavior [2], additional research is needed around the health implications of non-screen-based sedentary behavior [3]. This will help inform future updates of the guidelines to provide more specific recommendations around non-screen based sedentary behavior.

Despite the scientific evidence for the negative associations between screen time and cognitive development in the early years [2], previous qualitative [13-15] and quantitative studies [7,12] have reported that parents tend to believe that screen technologies are good learning tools. In the present study, even when the specific recommendations in the guidelines were presented around limiting screen time for children’s healthy growth and development, some parents still believed that screen-based activities were good for learning. However, an important finding of the present study was the value parents placed on cognitive development. Therefore, parents were eager for further information on the specific cognitive effects of screen time and admitted this might increase their motivation to adhere to the recommendations. Therefore, messages supplementing the guidelines and future interventions may be more effective if they focus on cognitive health outcomes versus physical health outcomes like obesity.

Regardless of parents’ attitudes towards sedentary behavior, parents unanimously expressed barriers to meeting the guidelines. The importance of the ‘electronic babysitter’ is consistent with findings from previous qualitative [13,15] and quantitative [6,7] work. A key finding of the present study was the desire and interest parents expressed for ideas and strategies for engaging children in non-sedentary and non-screen activities that could realistically keep children safe and occupied while they accomplished their daily tasks. This indicates supplementing the guidelines with resources that include alternative ideas and strategies may improve the adoption of the guidelines. Similarly, future intervention efforts may need to consider ways in which appropriate, specific, and practical strategies for alternative activities could be provided to parents. While not discussed in the focus groups, future work should consider using grandparents as a resource, since older generations engaged in child rearing in a world without tablets and smartphones. Furthermore, given the importance as well as the challenges acknowledged by parents in modeling healthy sedentary behavior habits, ideas and strategies for reducing parents’ and children’s sedentary behavior should also be considered.

Parental feedback on the communication of the guidelines to parents underlined the importance of receiving information about the guidelines as early as possible. The challenges expressed by parents in breaking sedentary behavior habits once formed is supported by the evidence that screen time habits formed at a young age tend to track overtime [8,9]. Many parents indicated they would like to receive this information during pregnancy through channels such as pediatricians’ offices and federal and provincial health agencies. This finding is informative for both the dissemination of the guidelines and the design and implementation of future interventions. Currently, few pediatricians in Canada (5%) are very familiar with the sedentary behaviour guidelines and only 27% almost always make recommendations around sedentary behavior to parents and caregivers of children in the early years [29]. Strategies are needed to increase awareness and reduce barriers for pediatricians in promoting the guidelines [29]. Equally informative was the guilty feelings parents described when the guidelines were presented to them. Therefore, consistent with evidence from the physical activity literature, messages around sedentary behavior for children of the early years may be most effective if they are framed in terms of gains versus losses [23].

Given the dearth of information in this area, the focus group questions were purposely not driven by a specific behavior-change theory. This allowed for the opportunity to examine whether a certain theory emerged from the findings. Results revealed that a wide range of interpersonal, social, and environmental factors influenced children’s sedentary behavior [30]. This suggests that a social-ecological model, such as the Ecologic Model of Sedentary Behavior [30], may be one appropriate framework for guiding future messages and intervention efforts. Additionally, Taylor, Baranowski, and Sallis’s Socialization Model of Child Behavior based on the Social Cognitive Theory may be another framework to consider [31]. In terms of children’s sedentary behavior, this theory suggests that parental behaviors, children’s cognitions/personal attributes, and the environment have a direct influence and parental cognitions have an indirect influence through parental behaviours and the environment. Parental behaviors also have an indirect influence through the environment [31]. Therefore, the Socialization Model of Child Behavior incorporates parental cognitions and behaviors as well as other important components identified in the focus groups, such as the environment.

A strength of this study was the focus on Sedentary Behaviour Guidelines compared to just screen time use. Consequently, the findings provided practical recommendations for future dissemination strategies and future interventions utilizing the Canadian guidelines or guidelines in other countries. Due to the homogenous sample of parents (i.e., primarily educated mothers from two-parent homes), future research is needed in other demographics of parents, including fathers, single parents, as well as parents without a college/university degree, to explore parents’ perceptions of the Sedentary Behaviour Guidelines more explicitly.

## Conclusion

This study provided unique insight into the perceptions of parents on the new Canadian Sedentary Behaviour Guidelines for the Early Years to help inform what messages should supplement the guidelines when disseminating them to parents and when using the guidelines in behavior-change interventions to increase adoption. Overall the findings indicate that gain-framed messages around the role of screen-based and non-screen-based sedentary behavior for children’s cognitive and social development might be most effective. Furthermore, providing parents the guidelines early with resources for minimizing sedentary behavior should also be considered. Future research is needed in other demographic groups of parents to confirm these findings.

## Competing interests

The authors declare no competing interests.

## Authors’ contributions

VC and AL conceived the study and VC oversaw its conduct. All authors participated in the design of the study. MC moderated the focus group, analyzed the data, and interpreted the results. VC assisted with the data analysis. VC, TB, NH assisted with the interpretation of the results. VC and MC drafted the manuscript. TB, NH, AL revised the manuscript for important intellectual content. All authors read and approved the final manuscript.
